# Amino acid metabolism reprogramming: shedding new light on T cell anti-tumor immunity

**DOI:** 10.1186/s13046-023-02845-4

**Published:** 2023-11-03

**Authors:** Yue Zheng, Yiran Yao, Tongxin Ge, Shengfang Ge, Renbing Jia, Xin Song, Ai Zhuang

**Affiliations:** 1 Department of Ophthalmology, Ninth People’s Hospital, Shanghai JiaoTong University School of Medicine, Shanghai, 20025 P. R. China; 2 Shanghai Key Laboratory of Orbital Diseases and Ocular Oncology, Shanghai, 20025 P. R. China

**Keywords:** Amino acid, Metabolism reprogramming, Tumor microenvironment, T cell, Immunotherapy

## Abstract

Metabolic reprogramming of amino acids has been increasingly recognized to initiate and fuel tumorigenesis and survival. Therefore, there is emerging interest in the application of amino acid metabolic strategies in antitumor therapy. Tremendous efforts have been made to develop amino acid metabolic node interventions such as amino acid antagonists and targeting amino acid transporters, key enzymes of amino acid metabolism, and common downstream pathways of amino acid metabolism. In addition to playing an essential role in sustaining tumor growth, new technologies and studies has revealed amino acid metabolic reprograming to have wide implications in the regulation of antitumor immune responses. Specifically, extensive crosstalk between amino acid metabolism and T cell immunity has been reported. Tumor cells can inhibit T cell immunity by depleting amino acids in the microenvironment through nutrient competition, and toxic metabolites of amino acids can also inhibit T cell function. In addition, amino acids can interfere with T cells by regulating glucose and lipid metabolism. This crucial crosstalk inspires the exploitation of novel strategies of immunotherapy enhancement and combination, owing to the unprecedented benefits of immunotherapy and the limited population it can benefit. Herein, we review recent findings related to the crosstalk between amino acid metabolism and T cell immunity. We also describe possible approaches to intervene in amino acid metabolic pathways by targeting various signaling nodes. Novel efforts to combine with and unleash potential immunotherapy are also discussed. Hopefully, some strategies that take the lead in the pipeline may soon be used for the common good.

## Introduction

Multiple cell types coexist in the tumor microenvironment (TME), including tumor, stromal, and immune cells, with different metabolic requirements [1]. Owing to the limited nutrients in the TME, cancer cells must compete for nutrients with other cells. To meet increased bioenergy and biosynthetic demands, support rapid proliferation, and reduce oxidative stress required for proliferation and survival, cancer cells autonomously change their metabolic flux through various metabolic pathways, a process known as metabolism reprogramming [2]. Although these altered metabolic pathways are beneficial for cancer cells, they can also serve as therapeutic targets. For example, through depleting the supply of nutrients or designing drugs to target altered metabolic pathways in tumor cells [3].

As first observed by Otto Warburg, cancer cells prefer glycolysis to oxidative phosphorylation, even in the presence of oxygen [4]. Since then, the in-depth study of tumor metabolism reprogramming has increased. In addition to glucose, amino acids are important nutrients that not only provide carbon and nitrogen sources for the synthesis of biological macromolecules such as nucleotides, but their metabolites can also enter the tricarboxylic acid cycle (TCA cycle) to provide cells with adenosine-triphosphate (ATP). Biologists have long known that although cancer cells consume fewer amino acids than glucose, amino acids are indispensable source of nutrients. Amino acids, including glutamine, contribute approximately 30–50% of the carbon in tumor cells, whereas glucose contributes only 10–15% [5]. The difference in amino acid requirements between cancer and normal cells has led to attention being paid to amino acid depletion, which has rapidly translated into the progress of clinical applications. One of the most successful examples is L-asparaginase. It functions as a component of standard chemotherapy regimens for acute lymphoblastic leukemia (ALL) by depleting asparagine in tumor cells [6]. This is instructive for the study of amino acid metabolism.

Immunotherapy has attracted significant attention in tumor therapy in recent years. Immune checkpoint blockade (ICB) has been identified as a promising treatment option for malignancies [7], based on the finding that the programmed cell death protein 1 (PD-1)/programmed cell death 1 ligand 1 (PD-L1) and cytotoxic T lymphocyte-associated antigen-4 (CTLA-4) signaling pathways play a key role in tumor immune escape. At present, anti-PD-1/PD-L1 antibodies alone or in combination with anti-CTLA-4 antibodies or targeted therapies have become first-line therapies for a variety of cancers such as metastatic melanoma, lung cancer, head and neck cancer, and triple-negative breast cancer [8]. However, the response to treatment is limited to a subset of patients. With anti-PD-1 therapy, only approximately 31–44% of patients with advanced melanoma [9], 22–25% with renal cell carcinoma [10], and 19–20% with non-small cell lung cancer [11] achieved a lasting response. Furthermore, some patients reported immune-related adverse events that necessitated treatment discontinuation, which was remarkably high in 60% of patients treated with ipilimumab [12].

Another giant of immunotherapy, chimeric antigen receptor (CAR)-T cell therapy, was the first adoptive cell therapy to enter clinical translation and commercialization [13]. Since then, significant improvement has been achieved in patients with hematologic tumors, especially B-cell-derived malignancies. Subsequently, chimeric antigen receptor-natural killer (CAR-NK) cells entered the field. Adoptive cell therapy has two features that complement the limitations of ICB. First, engineered T cells require only a single treatment to achieve lasting benefits. Second, in the case of ALL, more than 90% of patients responded to CAR-T cells [14]. Although adoptive cell therapy has achieved great success in hematological malignancies, its use in solid tumors has not yielded similar results. Moreover, adoptive cell therapy is associated with clinical toxicity, leading to cytokine release syndrome and immune effector cell-associated neurotoxicity syndrome [15]. Cytokine release syndrome occurs in up to 77% of patients with ALL treated with CD19 CAR-T cells [16]. Neurotoxicity is another common toxicity which has been associated with CAR-T cells targeting CD19 or CD20 in hematological malignancies, and it was observed in more than 60% of CAR-T cell clinical trials [17].

**Fig. 1:**
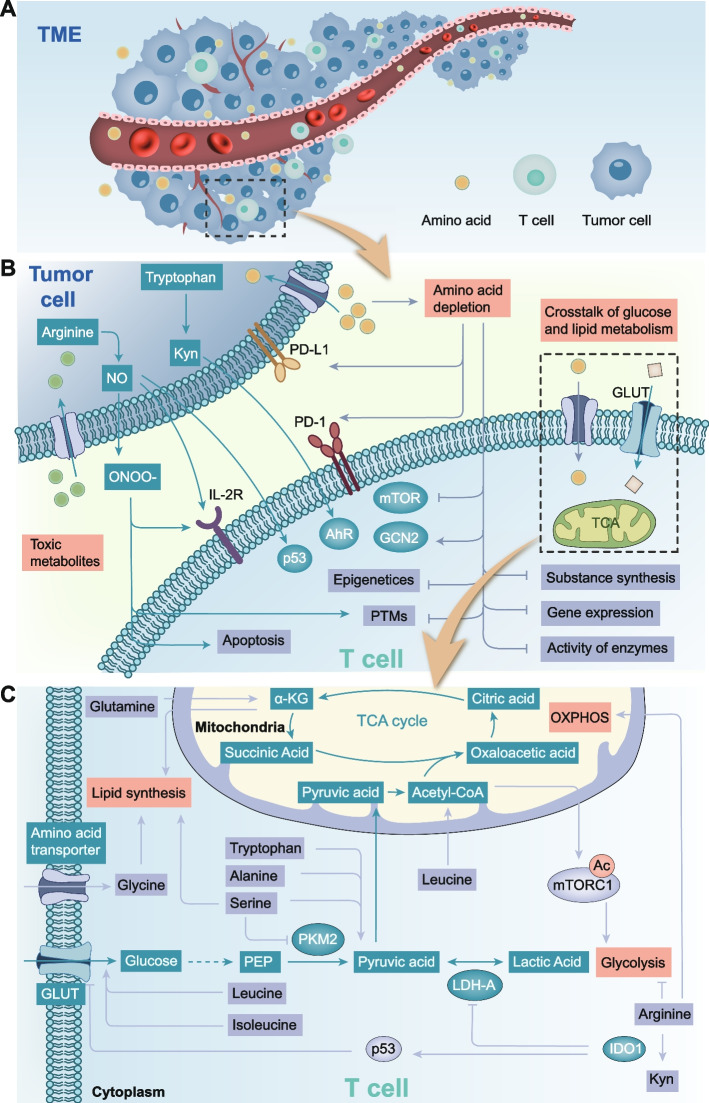
Schematic diagram summarizing the bridge between amino acid metabolism and T cell. **A** In the TME, T cells compete with tumor cells for amino acids. **B** Amino acid metabolism mainly affects T cell immunity through three aspects, including amino acid depletion caused by nutrient competition, toxic metabolites and crosstalk with glucose metabolism and lipid metabolism. Among them, amino acid depletion plays a role in T cell immunity through multiple mechanisms including mTOR, GCN2, PD-1/PD-L1, epigenetic and post-translational modifications. **C** Amino acid metabolism can affect glucose metabolism and lipid metabolism. On the one hand, amino acids regulate the activity of enzymes related to glucose metabolism or glucose transport. On the other hand, the intermediates of amino acid metabolism can directly act as substrates for glucose metabolism or lipid metabolism. Abbreviations: TME, tumor microenvironment; ONOO-, peroxynitrite; IL-2R, IL-2 receptor; PTMs, post-translational modifications; TCA cycle, tricarboxylic acid cycle; α-KG, α-ketoglutarate; OXPHOS, oxidative phosphorylation; GLUT, glucose transporter; PKM2, pyruvate kinase isozymes M2; PEP, phosphoenolpyruvate; LDH-A, lactate dehydrogenase A; IDO1, indoleamine 2, 3-dioxygenase 1

These limitations of immunotherapy suggest that the relationship between tumors and immunity is not fully understood. The key to immunotherapy is to activate the immune system to recognize and kill tumor cells. However, even during immunotherapy, there is no method to completely avoid tumor evasion of host immunity [18]. This is an important reason for the low response rates to immunotherapy. In recent years, the interaction between tumor cell metabolism and immunity has become the focus of our understanding of tumor immune evasion. Many studies have found that changes in amino acid metabolism can affect both tumor and T cells in the TME, leading to tumor immune escape (Fig. 1). In response to this finding, strategies targeting amino acids to enhance tumor immunotherapy have been proposed. A preclinical study has found that blocking CTLA-4 in conjunction with the inhibition of indoleamine 2, 3-dioxygenase (IDO), a key enzyme in tryptophan metabolism, significantly enhanced the antitumor effect of anti-CTLA-4 antibodies [19]. In addition, inhibitors of IDO combined with CAR-T cells can restore the control of IDO-positive tumors [20]. Therefore, combination immunotherapy with targeted amino acid metabolism is a promising strategy to enhance immunotherapy.

Given that the relationship between amino acid metabolism and T cells has not been thoroughly reviewed, we describe the crosstalk between amino acid metabolism and the immunosuppressive microenvironment as well as the feasibility and limitations of targeted amino acid metabolism therapy and combination therapy, which will contribute to the development of new cancer treatment strategies.

## Amino acid metabolism reprogramming in tumor cells abrogates T cell immunity

### Tumor cells inhibit T cell immunity through nutrient competition

**Fig. 2:**
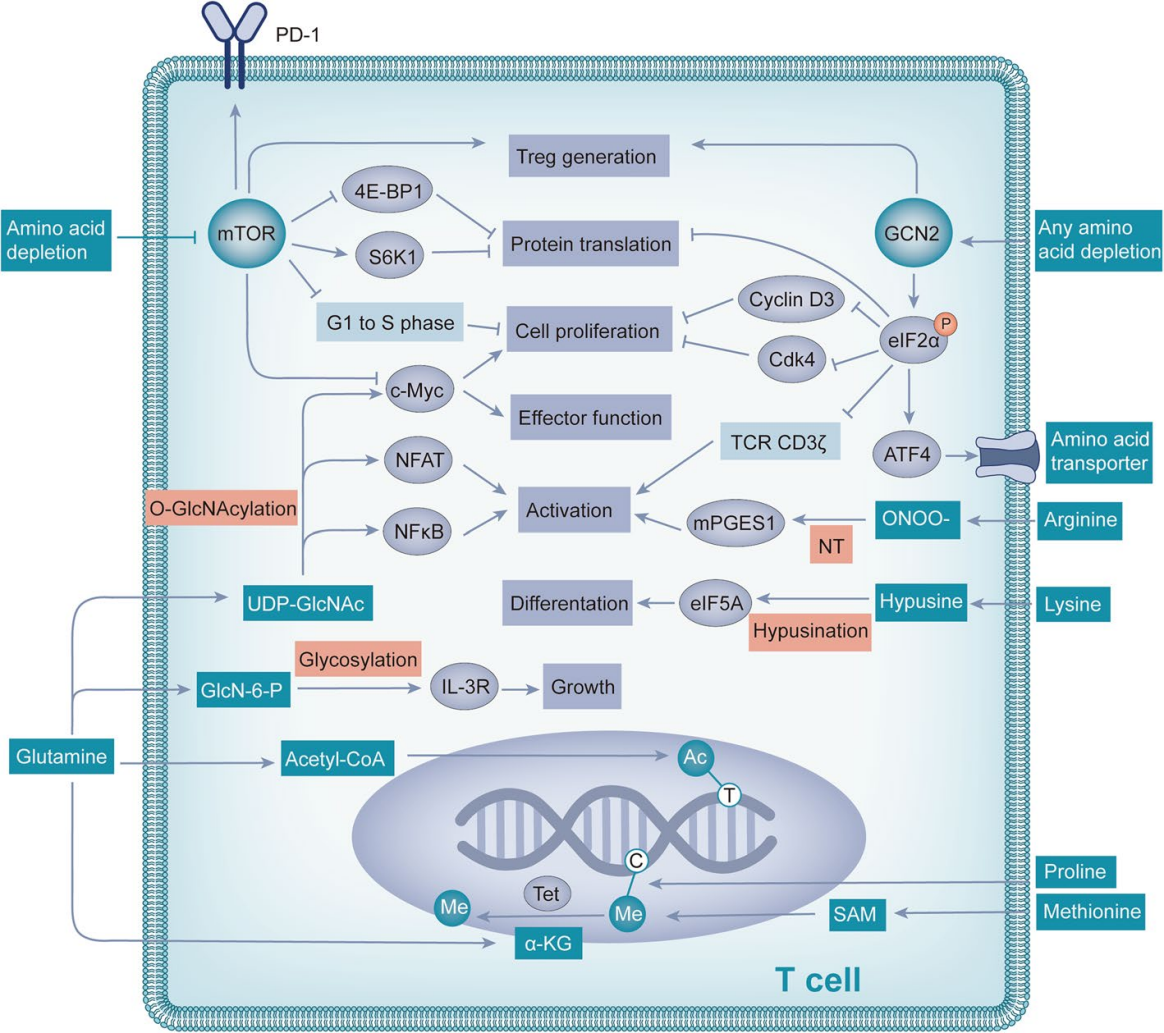
Nutrient competition affects T cells through a variety of mechanisms. Nutrient competition leads to amino acid depletion, which inhibits mTOR and activates GCN2, alters PD-1 expression, and affects epigenetic and post-translational modifications. Together, these pathways affect T cell protein translation, growth, proliferation, differentiation, activation, and effector function. Abbreviations: 4E-BP1, EIF4E-binding protein; S6K1, p70 S6 kinase; eIF2α, eukaryotic initiation factor 2; cdk4, cyclin-dependent kinase 4; NT, nitration of tyrosine; ONOO-, peroxynitrite; mPGES1, microsomal prostaglandin E synthetase 1; UDP-GlcNAc, uridine diphosphate n-acetyl glucosamine; IL-3R, IL-3 receptor; GlcN-6-P, glucosamine-6-phosphate; Ac, acetyl; T, Thymine; Me, methyl; C, Cytosine; SAM, S-adenosyl methionine; α-KG, α-ketoglutarate

In tumor tissues, owing to the high concentration of growth factors, the activation of key intracellular signaling molecules, such as c-Myc [21] and E2F [22] increases the expression of amino acid transporters, leading to the high uptake of amino acids by tumor cells and the depletion of amino acids. Nutrient limitation in the TME provides an environment in which immune, stromal, and cancer cells must compete for nutrients for biosynthesis, bioenergy, and effector functions. Immune cells are often not adapted to nutrient competition, which is the main mechanism regulating antitumor immunity [23]. T cells have received the most attention as the main tumor killer. The deleterious effects of arginine starvation on human T cells were first described in 1968 [24]. Arginine consumption has been found to lead to the inhibition of T cell activation under phytohemagglutinin stimulation. An increasing number of studies have shown that complex and diverse mechanisms are involved in the effects of amino acid starvation and T-cell immunity (Fig. 2).

#### Amino acid starvation regulates immune function through the mammalian target of rapamycin (mTOR) signaling pathway

Nutrient deficiency is a selective stress that shapes the evolution of most cellular processes [25]. Because amino acids play a crucial role in maintaining cellular homeostasis, different species have developed different mechanisms to detect amino acid abundance over the course of evolution. Eukaryotic cells are equipped with nutrient sensors such as mTOR, a conserved serine-threonine kinase that is activated when amino acids are abundant and regulates various anabolic processes required for growth [26]. Through binding with protein binding partners, mTOR can form mTOR complex 1 (mTORC1) or mTOR complex 2 (mTORC2) [27]. mTORC1 predominantly regulates metabolic reprogramming [28] and selectively regulates the differentiation of Th1, Th17 and cytotoxic CD8 + T cells [29]. mTORC2 mainly regulates actin polarization and endocytosis [30], and is closely associated with differentiation of Th2 and memory CD8 + T cell and migration of regulatory T cells (Tregs) [31, 32]. In conclusion, as the core component of mTORC1 and mTORC2, mTOR plays a key role in amino acid metabolism and immunity [33]. Not all amino acids can regulate mTOR activity; only leucine, arginine, lysine, glutamine, methionine, and tryptophan can regulate mTOR activity [34–38]. Inhibition of mTOR affects protein translation, cell proliferation, differentiation, effector functions, and many other factors.

Protein translation is regulated by mTOR through two independent mechanisms: inactivation of the EIF4E-binding protein (4E-BP1) and activation of p70 S6 kinase (S6K1) [39]. Ornithine decarboxylase (ODC) translation is controlled by the mTOR/4E-BP1 axis [40]. Polyamine metabolism is affected by mTOR by influencing ODC translation, which plays an important role in T cell activation and differentiation [41].

T cell proliferation is regulated by mTOR through two pathways. On the one hand, inhibition of mTOR activity impairs the activation of the c-Myc signaling pathway, leading to metabolic stress and defective T cell proliferation [42]. On the other hand, mTOR forms an intracellular complex with the serine-threonine kinase aurora B and survivin from the costimulatory molecule CD28, which is responsible for allowing the G1-S transition in antigen-stimulated T cells [43].

Inhibition of mTOR promotes differentiation of T cells into immunosuppressive Tregs. One Study has shown that mTOR inhibition promotes Treg production via Rag and Rheb GTPases [35]. Another study demonstrated that mTOR inhibition promotes the differentiation of naive CD4 + T cells into Foxp3 + Treg cells, which have a suppressive function in vivo, whereas mTOR signaling activation supports the differentiation of naive cells into Th1 cells [44]. In conclusion, mTOR inhibition induced by amino acid deprivation shifts the balance between Th1 and Treg production toward the Treg phenotype.

#### Amino acid starvation regulates immune function through the general control nonderepressible 2 (GCN2) signaling pathway

GCN2 is another eukaryotic amino acid sensor that, unlike mTOR, directly senses the depletion of individual essential or nonessential amino acids in cells by binding to uncharged cognate tRNAs [45]. In eukaryotes, the GCN2 and mTOR pathways are major regulatory switches that determine protein synthesis in response to fluctuations in amino acid levels [26].

In contrast to the mTOR regulatory mechanism, the absence of any amino acids activates GCN2 kinase activity [36]. As eukaryotic amino acid sensors, GCN2 and mTOR have similar effects on T cell function. GCN2 also regulates protein translation, proliferation, differentiation, and effector functions of T cells.

The effect of GCN2 activation is mainly mediated by phosphorylation of the downstream target eukaryotic initiation factor 2 (eIF2α). Amino acid depletion triggers signaling through GCN2 kinase and inhibits cyclin D3 [46] and cyclin-dependent kinase 4 (CDK4) through eIF2α phosphorylation, leading to reduced Rb protein phosphorylation, low E2F1 expression, and cell cycle arrest [47, 48]. However, phosphorylated eIF2α leads to downregulation of T-cell receptor (TCR) CD3ζ in CD8 + T cells [49, 50], which further leads to downregulation of Jak-3 and decreased translocation of NFκB-p65, ultimately impairing the proliferation and interferon-γ (IFN-γ) production of T cells [51]. Furthermore, phosphorylated eIF2α fails to bind to methionyl tRNA, which blocks the translation initiation of most mRNAs [52], but selectively enhances the translation of a few transcripts, such as activating transcription factor 4 (ATF4) [36]. Specifically for T cells, ATF4 promotes metabolism reprogramming of T cells, including upregulation of glycolysis, oxidative phosphorylation, and glutaminolysis, thus providing substrates and energy for anabolism [53]. The increase in ATF4 translation has a protective effect on T cells and partially neutralizes the adverse effects of amino acid depletion on T cells to a certain extent.

In addition to cell cycle arrest and effector impairment, GCN2 activation blocks Th17 differentiation [54] and drives de novo differentiation of Foxp3 + Tregs [55]. Furthermore, GCN2 kinase directly activates mature Tregs, which in turn exert immunosuppressive effects in a PD-1/PD-L1-dependent manner [56]. In conclusion, GCN2 appears to promote formation and immunosuppressive activity of Tregs, as well as inhibit effector T cells.

#### Amino acid starvation regulates immune function through PD-1/PD-L1

PD-1 and PD-L1 are extensively studied immune checkpoints. PD-1 is expressed on T cells, and its cognate ligand PD-L1 is expressed on target cells such as cancer cells. Binding of PD-1 to PD-L1 leads to inhibition of TCR-related signaling molecules, resulting in T cell depletion and protection of target tissues from T cell-mediated damage [57]. A variety of metabolic processes have been found to regulate the expression of PD-1/PD-L1 in the TME, such as glucose [58] and prostaglandin E2 (PGE2) [59] metabolism. Amino acid metabolism also affects the expression of PD-1/PD-L1.

Different amino acids regulate PD-1/PD-L1 through different mechanisms. Under glutamine restriction, the level of intracellular glutathione (GSH) decreased, which led to the upregulation of PD-L1 in tumor cells, and then inhibited T cell activity [60]. The relationship between glutamine and PD-L1 is also demonstrated by the fact that upregulated PD-L1 can return to normal levels after glutamine recovery [61].

Dendritic cells (DCs) expressing IDO, a key enzyme in the tryptophan metabolism pathway, can activate Tregs, which in turn upregulates the expression of PD-L1 on DCs and enhances the immunosuppressive effect of Tregs through the PD-1/PD-L1 interaction [56, 62]. Indoleamine 2, 3-dioxygenase inhibitors were found to activate CD8 + T cells and downregulate PD-1 expression by increasing tryptophan levels and degrading the PD-1 transcriptional activator NFATc1 [63].

In addition, multiple amino acid deletions inhibit mTOR activity and Akt phosphorylation, leading to Forkhead box O (FOXO) transcription factor activation and upregulation of PD-1 expression in Tregs. Subsequently, PD-1 binds to the ligand, activates the lipid phosphatase phosphatase with tensin homology (PTEN) in Tregs, inhibits phosphatidylinositol 3-kinase (PI3K) activity, and blocks phosphorylation at another Akt activation site to maintain Akt inhibition, forming a feedback loop [64]. This initiates a stable state of self-sustaining inhibition in Tregs, which is maintained by the circulation between PD-1 and Akt, leading to the sustained suppression of antitumor immunity.

In conclusion, amino acid depletion can lead to the upregulation of PD-L1 and PD-1 thereby inhibiting antitumor immunity.

#### Amino acid starvation regulates immune function through epigenetic and post-translational modifications (PTMs)

To prevent aberrant signaling that may adversely affect cell homeostasis, protein expression and activity must be strictly regulated. These regulatory mechanisms include epigenetic modifications of the genome and PTMs of proteins that determine the translational ability of transcripts and function of proteins, respectively. The core of epigenetics is the modification of histones and nucleic acids, which together regulate chromatin structure and gene expression, and produce genetic phenotypic changes without altering the DNA sequence [65]. Downstream of epigenetics is an additional level of regulation called PTM, which allows for the most refined and dynamic control of protein biology, including localization, conformation, interaction, and activation [66]. Amino acids are involved in a variety of epigenetic processes and PTMs associated with T-cell immunity (Table 1).

**Table 1:** Amino acids and metabolites involved in epigenetics and PTMs related to T cell immunity

Amino acid	Metabolic intermediates	Epigenetic modifications	Ref
Methionine	SAM	Methylation	[37]
Proline	/	Methylation	[67]
Glutamine	α-KG	Demethylation	[68]
Glutamine	Acetyl-CoA	Acetylation	[37]
BCAAs	Acetyl-CoA	Acetylation	[37]
		**PTMs**	
Lysine	Hypusine	Hypusination	[69]
Arginine	ONOO-	Nitrosylation of thiol	[70]
Arginine	ONOO-	Nitration of tyrosine	[70]
Glutamine	UDP-GlcNAc	O-GlcNAcylation	[38]
Glutamine	Glucosamine 6-phosphate	Glycosylation	[71]

*Abbreviations*: *PTMs* post-translational modifications, *SAM* S-adenosyl methionine, *α-KG* α-ketoglutarate, *BCAAs* branched-chain amino acids, *ONOO-* peroxynitrite, *UDP-GlcNAc* uridine diphosphate n-acetyl glucosamine

The main epigenetic modifications involved in amino acid metabolism are methylation, demethylation, and acetylation. The methylation of DNA and histones is dependent on methionine because its metabolite S-adenosyl methionine (SAM) is a universal methyl donor [37]. Loss of SAM in cells results in decreased Th17 polarization and death of CD8 + T cells [72]. Proline plays a role in epigenetic regulation by inducing specific histone methylation patterns [67]. DNA methylation can be reversed by removal of oxidized methylated bases by Tet proteins, a process that requires α-ketoglutaric acid (α-KG) [68]. α-KG is a TCA cycle intermediate, and glutamine is the main source of α-KG when glucose is scarce [73]. A recent study found that glutamine depletion is accompanied by a decrease in α-KG, which inhibits histone demethylation [74]. Histone acetyltransferases use acetyl-CoA to provide acetyl groups for acetylation. Glutamine and branch chain amino acids can be metabolized to produce acetyl-CoA [37]. The absence of these amino acids affects important cellular signaling pathways.

PTMs involving amino acids include hypusination, nitrosylation, O-GlcNAcylation, and glycosylation. These modifications modulate immune processes through various mechanisms, including regulating the activity of enzymes or signaling molecules, altering protein interactions, determining subcellular localization, and controlling protein translation.

Hypusine is a modified lysine formed by the reaction of lysine with spermidine [75]. SAM generated from methionine can be further transformed into spermidine, and hypusination mediated by it occurs only on the translation initiation factor eukaryotic initiation factor 5A (eIF5A) [69]. As a highly conserved protein, eIF5A is required for the elongation of the translation of specific mRNA transcripts and affects protein expression in a variety of immune cells [76]. Activation of eIF5A is required for the differentiation of CD4 + T cell subsets, and eIF5A inhibition leads to the selective reduction of Th1 cells [77].

Arginine metabolism produces nitric oxide (NO), which further reacts with superoxide to form peroxynitrite (ONOO^−^), which rapidly results in two PTMs on proteins: nitrosylation of thiol and nitration of tyrosine [59, 70]. One study has found that ONOO^−^ modifies chemokines such as CCL2 by nitrification or nitrosylation to inhibit T cell infiltration in tumor tissues [78]. In cells, ONOO^−^ has been found to regulate microsomal prostaglandin E synthetase 1 activity through post-translational nitration of tyrosine modification and positively regulate PGE2 production, which mediates the stimulation of immunosuppressive Tregs [59].

Glucose and glutamine can be metabolized to uridine diphosphate n-acetyl glucosamine (UDP-GlcNAc), a substrate for O-GlcNAcylation. O-GlcNAcylation is one of the most abundant PTMs [38]. Many signaling molecules regulated by O-GlcNAcylation are fundamental to T cell survival and biological function, such as c-Myc, NFAT, and NF-κB [38, 79, 80]. Among them, c-Myc plays a crucial role in the clonal expansion and effector function of T cells [81], whereas NFAT [82] and NF-κB [83] signal transduction are key regulators of T-cell activation.

Glutamine is required for N-linked glycosylation and is essential for protein stability and function. Aberrant glycosylation has been observed to affect cell proliferation and growth, as in the IL-3 receptor, where abnormal branching of the sugar chain leads to altered downstream signaling [71]. The depletion of amino acids leads to the weakening of glycosylation of proteins and lipids, thus affecting the function of many proteins.

#### Direct effects of amino acid starvation

There is increasing recognition that in addition to synthesizing proteins and peptides, some amino acids can act as signaling molecules and regulate key metabolic pathways that are necessary for immunity [84]. When amino acids are depleted, these biological processes can be directly inhibited, resulting in a disordered cell structure and function.

Amino acids are involved in the synthesis of many substances such as nucleotides, SAM, and GSH. Methionine is mainly involved in SAM synthesis and regulates epigenetic inheritance. Serine and glutamine are also involved in nucleotide synthesis. Serine is an important substrate for purine synthesis, and the entry of serine into one-carbon unit metabolism is a checkpoint for CD8 + T cell proliferation [85, 86]. Unlike serine, glutamine restriction mainly affects the CD4 + T cell subgroup. Glutamine depletion promotes differentiation of CD4 + T cells into Foxp3 + Treg cells by reducing nucleotide synthesis [87]. GSH is the most abundant antioxidant, and is synthesized from glycine, glutamate, and cysteine. Cysteine is a rate-limiting substrate of GSH synthesis [37]. GSH deficiency increases reactive oxygen species (ROS) and disrupts intracellular redox homeostasis, thereby affecting cell survival and function. Because of the different degrees of oxidative stress in Tregs and Th17 cells, GSH deficiency can promote Treg differentiation and inhibit Th17 differentiation, resulting in an imbalance between Treg and Th17 differentiation [88].

Amino acid deficiency can directly affect the expression levels of genes and downstream proteins that are closely related to the function of T cells. One previous study showed that T cells exhibit glutamine-dependent expression of cell surface activation markers CD25, CD45RO, and CD71, as well as IFN-γ and TNF-α production [89]. Besides this, glutamine deficiency in hepatocellular carcinoma upregulates the expression of *LAG3* and induces functional failure of γδT cells, which are involved in mediating antitumor responses and are associated with positive prognosis [90].

Immune cell proliferation and function occur through activation of key enzymes and proteins. Amino acid deficiency or some amino acid metabolizing enzymes can also directly affect the activity of these proteins or enzymes. Glutamine depletion leads to decreased activity of extracellular signal-regulated kinase (ERK) and c-Jun amino-terminal kinase (JNK) kinases, which further results in inhibited transcription of proliferation-related genes [91]. Arginine deprivation reduces F-actin content and CD2 and CD3 accumulation in T cell immune synapses by impinging on cofilin dephosphorylation, ultimately reducing proliferation and cytokine synthesis [92]. In addition, the tryptophan-metabolizing enzyme IDO can selectively reduce the activity of electron transport chain complex I, limiting ATP production in CD8 + T cells and leading to the inhibition of effector functions [93].

### Toxic metabolites of amino acids

High consumption of amino acids by tumor cells is accompanied by the production of toxic metabolites, which have been shown to exert inhibitory effects on T cell immunity.

#### Tryptophan metabolite kynurenine (Kyn)

In tumor cells, more than 95% of tryptophan is degraded via the Kyn pathway [94]. This ultimately leads to the biosynthesis of the cofactor nicotinamide adenine dinucleotide (NAD) [95]. This metabolic pathway produces metabolites, including Kyn, 3-hydroxykynurenine (3-HK), 3-hydroxyanthranilic acid (3-HAA), and quinolinic acid, all of which are collectively known as kynurenines. Among them, Kyn is the most widely studied protein associated with antitumor immunity. Tryptophan is catalyzed to Kyn by IDO1, IDO2, or tryptophan 2, 3-dioxygenase (TDO), and is the rate-limiting enzyme in this process [96]. Because IDO1 has the highest expression level and activity, many studies have identified IDO1 as a major cause of tryptophan depletion [97, 98].

On the one hand, Kyn can directly produce toxic effects on immune cells, inhibit the proliferation of T cells and induce their apoptosis. By arresting the cell cycle in the middle of the G1 phase, Kyn selectively inhibits proliferation of activated T cells, whereas resting T cells are unaffected and subsequently activate normally [99]. This inhibitory effect on T cell proliferation is concentration-dependent [100]. Simultaneously, Kyn can lead to changes in the intracellular redox balance and induce cell apoptosis through ROS production [101].

On the other hand, Kyn, as an endogenous aryl hydrocarbon receptor (AhR) agonist, contributes to immunosuppression of the TME and supports tumor immune escape. Kyn combined with AhR promotes the differentiation of CD4 + T cells into Tregs and inhibits the function of effector T cells [102, 103]. In Th17 cells, AhR activation promotes downstream IL-22 production and differentiation [104]. In addition, activated AhR controls the transcriptional program associated with tolerant DCs [105], which in turn inhibits T-cell immune activity. Activated AhR can also up-regulate the expression of PD-1 in CD8 + T cells [106, 107], inhibit T cell activity, and promote immune tolerance.

Of note, many in vitro experiments have used much higher concentrations of Kyn than the actual in vivo concentrations. The concentration of Kyn in human tumors is only in the low micromolar range, well below the 1 mM required to induce T cell apoptosis in vitro [108], which calls into question its clinical significance. However, treatment of mice with specific Kyn-depleting enzymes improved tumor growth and enhanced immunotherapy, suggesting that in vivo concentrations of Kyn-depleting enzymes may still be clinically relevant [98]. Further in vivo experiments are needed to verify the specific effects of Kyn.

Other products of the Kyn metabolic pathway, such as 3-HAA and 3-HK, also have immunosuppressive effects. 3-HAA inhibits T cell proliferation by suppressing the activation of pyruvate dehydrogenase and NF-κB [109], and impairs T cell activation by inhibiting TCR-mediated Ca^2+^ signaling in T cells [110]. In addition, 3-HAA stimulated TGF-β production and promoted Treg formation [111]. Notably, 3-HAA can induce selective apoptosis of Th1 cells by promoting the release of cytochrome C and activation of caspase 8 [112, 113] and mediate apoptosis via ROS production [114]. Moreover, 3-HK is also thought to reduce CD8 + T cell proliferation and maturation of naive T cells into CD8 + T cells [115].

#### Arginine metabolites NO and reactive nitrogen species

There are two main arginine metabolic pathways: arginine/Arginase (ARG)/ODC/polyamine and arginine/ inducible nitric oxide synthase (iNOS)/NO [116]. On the one hand, arginine produces ornithine through the urea cycle, and ornithine is used to synthesize polyamines, which are important for T cell growth [37]. On the other hand, arginine forms NO through iNOS, which further combines with O_2_^−^ to produce reactive nitrogen species, such as ONOO^−^ [117].

NO can block T cell function by interfering with the IL-2 receptor signaling pathway, thereby preventing the activation of multiple signaling molecules, including STAT5, Erk, and Akt [118]. In addition, NO can induce CD4 + T cells to differentiate into CD4 + Foxp3 + Treg cells through the NO/p53/IL-2/OX40/survivin signaling pathway, and iNOS inhibition completely inhibits NO-induced differentiation [119].

As mentioned in Sect. " Amino acid starvation regulates immune function through epigenetic and post-translational modifications (PTMs)", ONOO^−^ can regulate the immune response through PTMs. In addition, owing to its strong oxidizing effect, it can inhibit the activation-induced protein tyrosine phosphorylation or through nitration, inhibit a component of the mitochondrial permeability transition pore causing the release of cytochrome C and other death promoting factors, resulting in T cell apoptosis induction [120]. ONOO^−^ can also lead to changes in the expression of TCR, IL-2R, and CD8 molecules, thus damaging the T cell signaling pathway and inhibiting its effector function [78].

#### Other toxic metabolites

In addition to the above amino acid toxic metabolites, a recent study also found that the glycine derivative metabolite methylglyoxal can enter CD8 + T cells and combine with arginine. The consumption of free L-arginine and the destruction of the modification of proteins containing L-arginine can greatly inhibit the activation and function of CD8 + T cells [121]. In addition, cancer cells with abnormal glutamate decarboxylase 1 expression can use glutamine to synthesize gamma-aminobutyric acid (GABA) [122]. A main function of GABA is as an important neurotransmitter; in tumor tissues, GABA can activate the GABAB receptor and inhibit the activity of GSK-3β, resulting in enhanced β-catenin signaling [122]. Such GABA-mediated β-catenin activation can stimulate tumor cell proliferation and inhibit the intratumoral invasion of CD8 + T cells [122].

In conclusion, amino acid metabolism plays a significant negative regulatory role in T cell immunity. On the one hand, amino acid depletion inhibits immune cell function in many aspects; on the other hand, some toxic metabolites accumulate in the TME, further inhibiting T cell immunity and causing immune tolerance.

### Amino acid metabolism affects T cell immunity through the regulation of glucose and lipid metabolism

Different subgroups of T cells depend on different metabolic pathways and the metabolism of T cells in different states is not the same. CD8 + T cells require a high intake of glucose, amino acids, and fatty acids, and Tregs mainly use fatty acids for oxidation. Generation of CD8 + memory T cells depends on adequate fatty acid oxidation and require endogenous acetate supply [85]. Compared to resting T cells, after activation, glycolysis, pentose phosphorylation, and glutamine decomposition increased and fatty acid oxidation decreased in T cells [123]. Specifically for glucose metabolism, activated T cells show a relatively reduced dependence on oxidative phosphorylation (OXPHOS) and an increased need for glycolysis [124]. Thus, a normal and desirable metabolic state is essential for T cells to function. Amino acid metabolism can regulate the metabolism of other nutrients such as fat and sugar, ultimately leading to changes in the overall metabolism of T cells and inhibition of function.

Amino acid metabolism has the most significant effect on glucose metabolism. Metabolized amino acids can be converted into substrates in the glucose metabolic pathway to regulate glucose metabolism directly. The process can also regulate the enzyme activity of the glucose metabolic pathway and glucose transporters to have an indirect impact on glucose metabolism. A direct effect is achieved through the TCA cycle. Amino acids (such as alanine, tryptophan, and serine) can be converted to pyruvate, which partly promotes glycolysis and produces lactic acid for quick energy production [125].

Indirect regulation is a complex process. Many amino acids regulate sugar metabolism. Serine is an allosteric activator of pyruvate kinase isozymes M2 (PKM2) and supports aerobic glycolysis and lactic acid production by binding and activating PKM2 [124], which is essential for T cell function. Leucine and isoleucine were found to boost glucose uptake by increasing cell-surface glucose transporters [126]. In addition, leucine can be converted to acetyl-CoA, which can acetylate and activate mTORC1, further enhancing glycolysis [127]. The increase in arginine concentration promoted gluconeogenesis and the TCA cycle, whereas downregulated glucose transporters and glycolytic enzymes. These changes promote T cell OXPHOS and downregulate T cell activation-dependent glycolysis [128]. In addition, the tryptophan-metabolizing enzyme, IDO1, induces p53 expression and then inhibits glucose transporters and glycolysis [129]. At the same time, IDO1 inhibits lactate production by decreasing lactate dehydrogenase A (LDH-A) levels [129]. Also through the downregulation of glutaminase 2 (GLS2), IDO1 blocks the supply of glutamate, a substrate for the TCA cycle, making T cells more starved [129]. These changes ultimately inhibited T cell proliferation.

In addition to affecting glucose metabolism, amino acids regulate lipid metabolism. Glutamine metabolism produces α-KG, which is involved in fatty acid synthesis. Inhibition of glutamine uptake has been shown to reduce fatty acid synthesis and basal oxygen consumption [130]. Serine and glycine are necessary precursors for the synthesis of lipids [131], which are essential for cell growth, because the rapid proliferation of activated T cells relies on lipids to provide cell membranes.

**Fig. 3:**
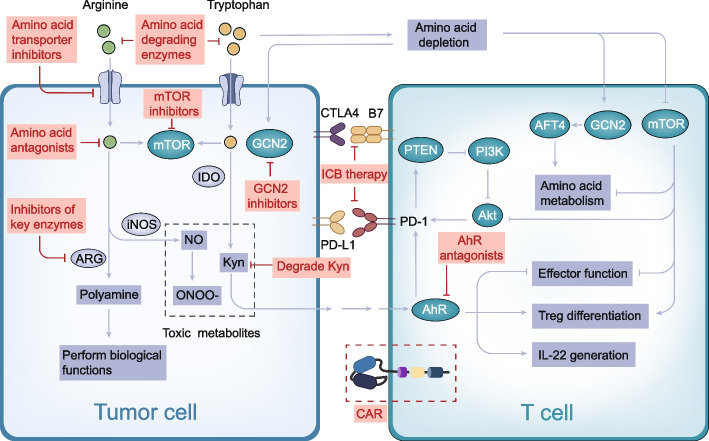
The mechanism diagram illustrates targets for targeted amino acid metabolism and immunotherapy. Amino acid degrading enzymes can deplete extracellular amino acid pools. Amino acid transport inhibitors inhibit amino acid transport into cells. Amino acid antagonists and inhibitors of amino acid metabolizing enzymes can inhibit amino acid metabolism and exert biological functions. Kyn degrading enzyme and AhR antagonist inhibit the toxic effects of Kyn on T cells from upstream and downstream, respectively. mTOR or GCN2 inhibitors can enhance efficacy in combination with drugs that target amino acid metabolism. The use of amino acid metabolism in ICB or CAR-T therapy can enhance immunotherapy efficacy. Abbreviations: ARG, arginase; iNOS, inducible nitric oxide synthase; PTEN, phosphatqase and tensin homologue; PI3K, phosphatidylinositol-3-kinase; ONOO-, peroxynitrite; ICB, immune checkpoint block; CAR, chimeric antigen receptor

Considering the important role of amino acids in tumors and the significant differences in amino acid requirements between tumor and T cells, targeted amino acid metabolism is reasonable for tumor therapy. Various targeted amino acid therapy strategies have been proposed, including (1) depletion of extracellular amino acid pools to inhibit amino acid uptake, (2) inhibition of amino acid transporters to reduce intracellular amino acid transport, (3) use of amino acid antagonists or amino acid-metabolizing enzyme inhibitors to inhibit amino acid metabolism, and (4) decomposition of toxic metabolites or inhibition of their downstream pathways **(**Fig. 3) (Table 2). Among these, based on the first strategy, asparaginase has been successfully used in the treatment of ALL. In addition, a variety of drugs, such as the inhibitors of arginase, PEG-Arg I (BCT-100) [132] and ADI-PEG20 [133], are currently in phase III clinical trials and are expected to achieve clinical conversion soon.

**Table 2:** Drugs targeting amino acid metabolism therapy and their targets

Type	Target	Drug	Clinical phase	Ref
Amino acid degrading enzymes	Asparagine	ASNase	Clinical application	[72]
	Arginine	PEG-Arg I (BCT-100)	III	[132]
	Arginine	ADI-PEG20	III	[133]
	Cysteine	Cysteinase	Experimental	[134]
	Cysteine	Cysteinase-PEG	Experimental	[135]
	Cystine	Cystinase	Experimental	[136]
Amino acid transporter inhibitors	LAT1(SLC7A5)	LAT1-IN-1 (BCH)	Experimental	[137]
		JPH203	II	[138]
		SKN101	Experimental	[139]
	SLC1A5(ASCT2)	γ-L-glutamyl-p-nitroanilide (GPNA)	Experimental	[140]
		V-9302	Experimental	[141]
		Benzylserine (BenSer)	Experimental	[142]
	SLC6A14	α-methyltryptophan (α-MT)	Experimental	[143]
	SLC3A2	IGN523	I	ClinicalTrials.gov
	SLC7A11(xCT)	Sulfasalazine	I/II	ClinicalTrials.gov
Amino acid antagonists	Glutamine	6-diazo-5-oxo-L-norleucine (DON)	II	[144]
		JHU083	Experimental	[145]
Amino acid metabolic enzyme inhibitors	IDO	Indoximod (1-MT)	II	[146]
		NLG-802	I	ClinicalTrials.gov
		Epacadostat (INCB024360)	III	[147]
		NTRC 3883–0	Experimental	[148]
		BMS-986205	III	[149]
		KHK2455	I	ClinicalTrials.gov
		PCC0208009	Experimental	[150]
		PF-06840003	I	ClinicalTrials.gov
		GDC-0919	I	ClinicalTrials.gov
		MK-7162	I	ClinicalTrials.gov
		LY3381916	I	ClinicalTrials.gov
	IDO/TDO	Navoximod (NLG919)	I	[151]
		N-Benzyl/Aryl Substituted Tryptanthrin	Experimental	[152]
		pf06840003	I	ClinicalTrials.gov
		SHR9146	I	ClinicalTrials.gov
		DN1406131	I	ClinicalTrials.gov
		HTI-1090	I	ClinicalTrials.gov
	TDO	680C91	Experimental	[153]
		LM10	Experimental	[153]
	ARG	NOHA	Experimental	[154]
		nor NOHA	I	ClinicalTrials.gov
		CB-1158	I/II	ClinicalTrials.gov
	GLS	Telaglenastat (CB-839)	II	[155]
		BPTES	Experimental	[60]
		Glutaminase inhibitor 968	Experimental	[156]
	GDH	R162	Experimental	[157]
	PHGDH	CBR-5884	Experimental	[158]
		NCT-503	Experimental	[159]
Kyn/AhR pathway	Kyn	PEG-KYNase	Experimental	[160]
	AhR	IDB-AHRi	Experimental	[161]
		CH223191	Experimental	[162]

*Abbreviations*: *IDO* indoleamine 2, 3-dioxygenase, *TDO* tryptophan 2, 3-dioxygenase, *ARG* arginase, *GLS* glutaminase, *GDH* gutamate deaminase, *PHGDH* phosphate dehydrogenase, *Kyn* kynurenine, *AhR* aryl hydrocarbon receptor

Following the revelation of the complex relationship between amino acid metabolism and T cells, strategies for combining targeted amino acid metabolism with immunotherapy, including ICB and adoptive cell therapy, have been proposed **(**Fig. 3). Enhanced immunotherapy can be achieved in combination with the above amino acid-targeting agents or by reshaping the amino acid metabolism in CAR-T cells. To date, these studies have been conducted extensively, with several combination strategies already in clinical trials.

## Inspiration for anti-tumor therapy

### Therapeutics targeting amino acid metabolism

Because amino acids are critical, it is no surprise that cancer cells are highly dependent on the external supply of amino acids to maintain amino acid homeostasis. In contrast, owing to the increased nutritional requirements, tumor cells express higher levels of amino acid transporters and have higher amino acid metabolic activity than normal cells [163]. Thus, limiting the availability of amino acids should have specific adverse effects on tumor cells, whereas normal cells should remain mostly unaffected. Therefore, amino acid starvation therapy proposed on this basis is an attractive treatment option. Targeting amino acid metabolism in tumor cells is expected to disrupt the intracellular metabolic balance and tumor cytoskeleton, inhibit tumor cells, and relieve the inhibitory effect of amino acid depletion on T cells.

#### Use of amino acid degrading enzymes to deplete tumor amino acid pools

Leukemia cells are highly dependent on extracellular asparagine because of a deficiency in asparagine synthase, the only enzyme capable of asparagine synthesis [164]. L-asparaginase, which directly targets asparagine metabolism, has been successfully used in chemotherapy for ALL [72]. The successful application of asparaginase demonstrates the feasibility of targeting amino acid metabolism and is expected to promote the clinical transformation of metabolic enzyme-based amino acid deprivation therapy.

Similar to dependence of leukemia cells on exogenous asparagine, some solid tumors, including melanoma and hepatocellular carcinoma, depend on extracellular arginine for survival owing to the lack of de novo synthesis of arginine [165]. Therefore, two polyethylene glycol (PEG)-conjugated arginine decomposers have been developed to deplete the extracellular arginine pool. PEG functional modification can reduce its immunogenicity, prolong its blood circulation time and half-life in vivo, and improve its antitumor effect in vivo. PEG-Arg I (BCT-100) can convert arginine to ornithine, resulting in rapid depletion of extracellular and intracellular arginine libraries and reduced proliferation of tumor cells after monotherapy [166]. However, PEG-Arg I inhibited T cell proliferation and blocked T cell responses indirectly by inducing accumulation of bone marrow-derived suppressor cells (MDSCs). Therefore, L-arginine depletion therapy has a dual role in cancer therapy, with a risk of immunosuppression. ADI-PEG20, another drug that converts arginine to citcitine, has an unsatisfactory therapeutic effect because of the increased compensatory production of endogenous arginine caused by the overexpression of arginine succinic synthase 1 after monotherapy; however, it can achieve a certain effect in arginine succinic synthase 1-deficient tumor cells [136].

In addition, cystine or cysteine therapy leads to intracellular GSH depletion and ROS accumulation by increasing AMPK phosphorylation and decreasing mTOR phosphorylation, resulting in cell cycle arrest and cell death in various cancers [136].

#### Amino acid transporter inhibitors

Amino acid transmembrane transport is mediated by various amino acid transport systems within the solute carrier (SLC) superfamily [167]. More than 60 SLC proteins have been identified as amino acid transporters [168]. The relationship between amino acids and their transporters is complex. An amino acid can be transported by several different transporters, whereas a transporter passes through multiple amino acids [143]. In addition, tumor cells and immune cells have different expression levels of the same transporter [72]. In tumor cells, the imbalance of amino acid transporters leads to metabolism reprogramming, which changes the intracellular amino acid level and is an important mechanism leading to tumor development [168]. Therefore, amino acid transporters are reliable targets for tumor therapy.

Among the upregulated amino acid transporters in cancer cells, LAT1 (SLC7A5) is notable for cancer-specific expression. LAT1 transports almost all the neutral amino acids. BCH (2-aminobicyclo-(2,2,1)-heptane-2-carboxylic acid) was identified as an inhibitor of LAT1. It inhibited the proliferation of tumor cells in a dose-dependent manner. It also inhibited mTOR phosphorylation and induced cell cycle stasis in the G1 phase [137]. Another inhibitor of LAT1, JPH203 (KYT-0353), has a high affinity more than a thousand-fold higher than that of BCH [169]. JPH203 can lead to inhibition of the mTOR system, which leads to changes in downstream signaling pathways, in which cell cycle regulators such as cyclin-dependent kinase (CDK) 1–6 are considered to be the most downregulated kinases upstream [170]. JPH203 has shown encouraging results in Phase I clinical trials against advanced solid tumors and is currently being used in Phase II studies (UMIN000034080) [138, 171]. LAT1 is also unique in that its expression is tumor-specific and, therefore, can be used for the delivery of antitumor drugs. For example, because melphalan is transported by LAT1, antitumor L-phenylalanine mustard melphalan was designed to improve the cellular uptake of nitrogen mustard [169]. Similarly, the precursor of sesamol was designed by para-binding of sesamol to L-phenylalanine via a carbamate bond, which significantly enhanced its uptake and toxic effect on tumor cells [172]. In conclusion, the LAT1-mediated prodrug delivery strategy facilitates the selective uptake of drugs to increase their intracellular concentration and antiproliferative activity by targeting tumor cells that overexpress the LAT1 protein.

SLC1A5 (ASCT2) is the main glutamine transporter, and a variety of drugs have been developed for this transporter, such as γ-L-glutamyl-p-nitroanilide (GPNA), V-9302, and benzylserine. Selective pharmacological drugs have been developed based on the first-generation low-efficiency glutamine transport antagonist GPNA, and V-9302, a GPNA derivative, improved the ability to inhibit glutamine uptake in cells by approximately 100 times [173]. Blocking ASCT2 with V-9302 attenuates cancer cell growth and proliferation, and increases cell death and oxidative stress, which together promote antitumor responses [141]. Noteworthily, CD8 + T cells upregulate the glutamine transporter SLC6A14 via a compensatory pathway and maintain glutamine uptake and effector functions [28]. In other words, V-9302 selectively blocks the uptake of glutamine by tumor cells but not by CD8 + T cells and promotes the synthesis of the cellular antioxidant glutathione, thereby improving the efferent function of CD8 + T cells [174]. It has also been found that inhibiting glutamine metabolism with V-9302 may increase the expression of PD-L1 in tumor cells, thus inactivating T cells [60]. The ASCT2 inhibitor benzylserine can significantly reduce glutamine transport in tumor cells, inhibit the mTOR signaling pathway, and reduce the expression of cell cycle regulators, thus inhibiting cell cycle progression [142].

The amino acid transporter SLC6A14 transports all neutral amino acids, as well as the cationic amino acids lysine and arginine, and is a novel drug target. Alpha-methyltryptophan (α-MT), an inhibitor of this transporter, induces amino acid starvation and autophagy in tumor cells by blocking SLC6A14, inhibiting mTOR signal transduction, inducing amino acid starvation, and inhibiting tumor cell growth and proliferation [143].

SLC3A2 forms a heterodimer amino acid transporter with SLC7A5 or SLC7A11. Although SLC7A5 and SLC7A11 are actual transporters, they require SLC3A2 as a partner to recruit them into the plasma membrane [163]. Treatment with the humanized anti-SLC3A2 monoclonal antibody IGN523 showed antitumor efficacy in leukemia-derived and non-small cell lung cancer models [72]. IGN523 causes tumor cell death through NK cell-mediated cytotoxicity and inhibits the uptake of amino acids such as phenylalanine by tumor cells [175].

The cystine glutamate reverse transporter SLC7A11 (xCT) helps fight oxidative stress by promoting GSH-mediated antioxidant defenses. Therefore, xCT may be a promising target for cancer therapies. The xCT transport inhibitor sulfasalazine induced a decrease in cysteine and GSH and led to enhanced mitochondrial metabolism, resulting in increased ROS production, which triggered oxidative damage [176].

#### Use of amino acid antagonists to compete for amino acid metabolism

Glutamine antagonists have a long history of use. 6-Diazo-5-oxygen-L-deamine, a glutamine antagonist, inhibits glutamine-based enzymes [177]. In addition, two other compounds, acivicin and azaserine, are also glutamine antagonists [96]. However, owing to the important role of glutamine metabolism in normal tissue physiology, these compounds also cause varying levels of gastrointestinal toxicity, myelosuppression, and neurotoxicity, and were therefore deprecated [96].

The prodrug developed on this basis, JHU083, is a well-tolerated, brain-penetrating glutamine antagonist, and a promising new drug for treatment [178]. JHU083 can release 6-diazo-5-oxygen-L-deamine when cleaved by tumor cathepsin, thus playing a specific killing role in tumors [145]. Notably, this drug can differentially metabolize cancer cells and T cells, not only starving the cancer cells, but also making the TME a more suitable microenvironment for effector T cells, thus enhancing their attack on the tumors [179].

#### Inhibitors of key enzymes in amino acid metabolic pathways

##### Tryptophan

The tryptophan decomposing enzyme is frequently expressed in human tumors, which causes tumor cells to consume a large amount of tryptophan and produce many toxic products, resulting in immune suppression. Previous studies suggest that most human tumors constitutively express IDO, which is an important mechanism of tumor immune tolerance [180, 181]. In addition, tryptophan-decomposing enzyme activity is easily blocked by drug inhibitors [181]. Therefore, IDO/TDO inhibitors have been extensively studied.

Indoximod (1-MT) is the first IDO1 inhibitor to enter clinical development for cancer treatment [182]. It can eliminate Kyn production, inhibit tryptophan consumption, and restore T cell proliferation. The 1-MT prodrug NLG802 can significantly enhance the antitumor response of T cells [183]. It can be rapidly metabolized to 1-MT upon entry into the body, increasing its bioavailability five-fold, and has shown a safe toxicological profile at the intended therapeutic dose. Epacadostat is another selective inhibitor of IDO1 [184]. An in vitro experiment has shown that epacadostat promotes effector T cell growth and IFN-γ production and reduces the conversion to Tregs [185]. The novel small-molecule inhibitor NTRC 3883–0 can effectively counteract IDO1-induced changes in tryptophan and Kyn levels [148]. BMS-986205 is a highly effective and selective inhibitor of IDO1 that can effectively inhibit Kyn synthesis in IDO1-overexpressed cells [186].

Originally used as antidepressants, TDO inhibitors are also being explored as cancer treatments, such as 680C91 and LM10 [187]. As IDO and TDO are expressed differently in different tumor types, dual IDO/TDO inhibitors may be more advantageous. RG70099, for example, can reduce serum Kyn levels by approximately 90% [47]. Navoximod (NLG919) selectively inhibits IDO1 and TDO2, thereby reducing the proportion of Tregs and increasing T cell activation [188]. N-benzyl/aryl-substituted tryptanthrin, a dual inhibitor of IDO and TDO, can directly interact with IDO1, IDO2, and TDO to block the canine urine pathway and promote the proliferation of T cells, ultimately inhibiting the growth of tumor cells [152].

In addition to directly inhibiting IDO1, targeting the events upstream of IDO1 is an alternative strategy. A successful example of this approach is the tyrosine kinase inhibitor imatinib, which inhibits IDO1 expression [189]. Furthermore, induction of IDO1 in the TME can be prevented by inhibiting the activity of cyclooxygenase-2 (COX-2), a key enzyme for PGE2 production, which is capable of inducing IDO1 expression. A preclinical study has shown that COX-2 inhibition can reduce IDO1 levels and inhibit tumor growth and metastasis [190].

##### Arginine

Overexpression of ARG in MDSCs can lead to L-arginine deletion in the TME, thus inducing T cell apoptosis [117]. N-hydroxy-l-arginine (NOHA) is an ARG inhibitor that significantly inhibits ARG1 expression. It can restore the responsiveness of tumor-infiltrating T cells to stimulation, while inducing cell cycle arrest and apoptosis and reducing spermine production [154]. NOHA also inhibits the MDSC-mediated expansion of Tregs [191]. Nω-hydroxyl-non-arginine (nor NOHA) can eliminate stagnation of T cell proliferation and facilitate an immune attack against cancer cells [192]. CB-1158 is an ARG1 inhibitor. By inhibiting ARG, CB-1158 effectively blocks MDSC-mediated immunosuppression and reduces tumor growth by increasing the supply of arginine required for T cell proliferation [193].

##### Glutamine

Glutamine is converted to glutamate by GLS and glutamate is converted to α-KG by two types of reactions [194]. Transaminases convert amino groups from glutamic acid to ketoacids to produce α-KG and other amino acids. The other enzyme is glutamate deaminase (GDH), which releases ammonia and produces α-KG without consuming ketoacid. Glutaminase is a key target for glutamine metabolism, and GLS inhibitors have been used in various cancers [111]. There are three main GLS inhibitors: telaglenastat (CB-839), BPTES, and GLS inhibitor 968. CB-839 is an effective, selective, and reversible GLS inhibitor that allosterically inhibits the dimer-to-tetramer GLS transition, a key step in enzyme activation [195]. Inhibition of glutaminase by CB-839 significantly reduces the production of GSH, resulting in increased ROS and apoptosis [196]. However, another study found that CB-839 showed an early effect in pancreatic cancer cells, but the tumor cells soon adopted an adaptive metabolic network to maintain glutamine metabolism and proliferation in a GLS-independent manner [197]. Uncertainty regarding the efficacy of CB-839 suggests the need for continued mechanistic, pharmacological, and translational research [173]. Inhibition of glutamine metabolism by BPTES can increase the expression of PD-L1 in tumor cells, thus inactivating T cells [60]. Glutaminase inhibitor 968 functions as an allosteric GLS inhibitor [198] and has been found to have a good effect on tumor stem cells in glioblastoma and diminished tumor growth [156]. These findings highlight the importance of glutamine metabolism and support GLS as a therapeutic target for tumors.

Glutamate deaminase is also a target of glutamine metabolism. It has recently been reported that shRNA or GDH-specific inhibitor R162 targeting GDH leads to a significant reduction in α-KG and glutamine-dependent RNA biosynthesis, as well as an increase in ROS levels [157]. However, another study showed that the inhibition of GDH leads to increased cytoplasmic aspartate aminotransferase expression [199]. Thus, sufficient reducing power is generated to resist ROS and support cancer cell survival. These results suggest that targeting GDH alone may induce the activation of other metabolic pathways to reduce ROS and upregulate α-KG production, resulting in therapeutic tolerance.

##### Serine

In addition to the three most studied amino acids (glutamine, arginine, and tryptophan), recent studies have strongly suggested that tumor cells have a strong ability to synthesize serine de novo through the glycerol phosphate dehydrogenase (PHGDH) pathway. In a typical pathway for the synthesis of serine, PHGDH catalyzes the conversion of glycerate 3-phosphate produced during glycolysis to hydroxypyruvate 3-phosphate, the first rate-limiting step [96]. In addition, the reduction of serine by inhibiting PHGDH resulted in the inhibition of serine-based sphingolipid synthesis, an increase in bypass metabolic pathways, and the accumulation of the metabolite deoxysphingolipid, which has previously been reported as an anticancer factor [200]. Therefore, inhibition of PHGDH may play an antitumor role. Several PHGHD inhibitors have been identified. CBR-5884 can inhibit de novo serine synthesis in tumor cells and produce selective toxicity in cancer cell lines with high serine biosynthesis activity [158]. The small-molecule inhibitor NCT503 can also reduce serine production and inhibit the growth of cancer cells [159].

#### Targeting the Kyn/AhR pathway

In addition to inhibiting IDO/TDO, the key enzyme for Kyn production, other strategies targeting the Kyn/AhR pathway have been proposed, including decomposition of Kyn and inhibition of AhR.

PEG-KYNase is a recombinant enzyme that degrades Kyn into an immunoinert metabolite. PEG-KYNase has been shown to be therapeutic in multiple mouse models for tumor when used alone or in combination with checkpoint blocking [160]. Degradation of Kyn inhibits AhR activation. The modified kynureninase can degrade extracellular Kyn and has shown remarkable efficacy in mouse tumor models [201].

Aryl hydrocarbon receptor antagonists also attenuate immunosuppression and inhibit tumor growth. IDB-AhRi is an AhR antagonist that blocks the nuclear transposition of AhR and increases the expression of IFN-γ and TNF-α. IDB-AhRi increases tumor infiltration by CD8 + T cells and decreases Tregs and tumor-associated macrophages in mouse models of colon cancer [161]. Selective AhR inhibitors (CH223191) can block the immunosuppressive effects of Tregs [162], inhibit Th17 differentiation [202], and reduce PD-1 expression [106].

### Challenges in therapeutics targeting amino acid metabolism

Targeting amino acid metabolism is theoretically feasible, and preclinical and clinical trials of drugs are being carried out extensively. However, some challenges remain to be resolved. Drugs that target metabolism are typically administered systemically, which increases their potential toxic effects in normal tissues. Amino acids are important nutrients; therefore, blocking their metabolism can easily affect multiple organs of the body. This is why some of the previously studied drugs that target amino acid metabolism have many side effects in normal organs, such as the gastrointestinal tract.

Moreover, although amino acid metabolism is significantly elevated in many tumors, therapies targeting amino acid metabolism in patients with tumors have not yielded satisfactory therapeutic results. This may reflect the complexity of the TME. Our understanding of the metabolic interactions in this microenvironment is rudimentary, making it difficult to kill tumor cells without harming the antitumor immune cells. T cells also consume large amounts of glutamine when activated and proliferating [145]. Therefore, we need to consider whether depletion of the amino acid pool in the TME has a greater effect on tumor or T cells, as both cells benefit from the increase in local amino acids.

The third challenge is that the metabolism of tumor cells is plastic. The intracellular metabolism forms a complex network. When a node is blocked, cells can bypass it through compensatory mechanisms. Targeting an amino acid metabolic node alone may induce compensatory amino acid replenishment via other pathways. As mentioned earlier, pancreatic cancer cells have a compensatory metabolic network for GLS inhibitors [197]. After prolonged GLS inhibition, the tumor showed compensatory glutamine metabolism and growth recovery.

Finally, different tumors are dependent on different amino acids, and even the same tumor may exhibit different metabolic requirements, which increases the difficulty of targeting amino acid metabolism. For example, breast cancer cells show systemic differences in glutamine dependence, with basal cells favoring glutamine dependence and luminal cells favoring glutamine independence [203]. In addition, a high amino acid intake does not imply dependence. One previous study has shown that although luminal breast cancer cell lines consume almost the same amount of glutamine as triple negative breast cancer cells, the former are not sensitive to glutamine uptake inhibition [204]. Therefore, defining the metabolic characteristics of cancer subtypes is necessary to reveal how metabolic vulnerability can be exploited therapeutically [205].

Thus, before the clinical application of amino acid starvation therapy, not only should the metabolic dependence of specific cancer types be studied [206, 207] but the need for combination therapy in the face of this metabolic complexity should also be considered, which may be more effective.

### Targeting amino acid metabolism combined with mTOR/GCN2 inhibitors

In eukaryotes, mTOR and GCN2 are amino acid sensors, and the signaling pathways mediated by these two sensors are involved in the adaptive switching to alternative fuels when a certain metabolic pathway is inhibited. Therefore, combination therapies targeting amino acids and one of these two amino acid receptors have been proposed.

The increased metabolism of glutamine promotes its resistance to mTOR inhibition, and the expression of GLS increases after mTOR inhibition [208]. Therefore, the simultaneous use of glutaminase and mTOR inhibitors can achieve improved antitumor effects. There was a synergistic effect between CB-839 and mTOR inhibitor [173]. In addition, inhibiting glutamine metabolism into GSH combined with the mTOR inhibitor can enhance tumor cell death [209].

GCN2 inhibitors (GCN2iA) sensitize tumor cells to asparaginase by reducing the expression of asparagine synthase, thereby reducing de novo protein synthesis levels [210]. Another study showed that amino acid depletion therapy universally induced vascular endothelial growth factor expression through the GCN2/ATF4 pathway, and the inhibition of GCN2 reduced tumor vascular density [211].

### Targeting amino acid metabolism combined with immunotherapy

In recent years, immunotherapy has made great progress, and ICB and adoptive cell therapy have been used in clinical practice; however, a large proportion of patients do not benefit from immunotherapy. Amino acid metabolism plays an important role in T cell immunity. Therefore, immunotherapy combined with targeted amino acid metabolism may be a new direction for tumor treatment.

#### Targeted amino acid metabolism therapy combined with ICB therapy

The targeted therapy of amino acid metabolism combined with immunotherapy still mainly focuses on the three main amino acids, glutamine, arginine, and tryptophan. Glutamine transporter inhibitor V-9302 and GLS inhibitor BPTES, when used in combination with anti-PD-L1 antibodies, strongly promoted the effector function of T cells [60]. Another study found that the simultaneous transport of anti-PD-L1 antibodies and V9302 with molybdenum disulfide significantly promoted the infiltration of CD8 + T cells and strongly inhibited tumor growth [212]. Immunotherapy of the PD-1 checkpoint combined with glutamine-targeting JHU083 showed significantly increased response rates compared to those of PD-1 monotherapy [179]. These results demonstrate the correlation between tumor glutamine metabolism and antitumor immunity and suggest that the combined targeting of glutamine metabolism and PD-L1 is a promising therapeutic approach that can significantly enhance the antitumor effect.

CB-1158, an ARG1 inhibitor, showed a highly potent antitumor effect when used in combination with anti-PD-1 antibodies or anti-CTLA-4 antibodies [193]. In addition, the subtype of arginase expressed by T cells, mitochondrial ARG2, can regulate T cell activation, antitumor cytotoxicity, and memory formation in CD8 + T cells, independent of extracellular arginine availability. In addition, the specific loss of ARG2 in CD8 + T cells has a strong synergistic effect with PD-1 blocking in tumor growth control [213]. Inhibition of ARG2 in conjunction with PD-1 blocking therapy may improve the response to immunotherapy.

One Study has shown that plasma Kyn:Trp ratio increases in patients with tumors during pembrolizumab treatment [107]. Another preclinical study showed that the IDO inhibitor 1-MT enhanced the efficacy of anti-CTLA-4 and anti-PD-1/PD-L1 treatments by increasing the infiltration and activation of CD8 + T cells at the tumor site [19]. These findings suggest that the high expression level of IDO and the corresponding increase in Kyn may be the underlying factors that induce tolerance to ICB therapy. In addition, numerous experiments have demonstrated that tumor cells expressing IDO can suppress immune cells through tryptophan starvation and that AhR is involved in tumor immune escape. Therefore, combination therapy targeting the Trp-Kyn-AhR axis in immunotherapy has strong translational rationality and good preclinical effects; however, the clinical trial effect of combination therapy is not satisfactory [214] and has reignited the combinatorial approach debate. This may be because of the role of TDO in immune escape, although its role in tumors is not as important as that of IDO. Therefore, the dual inhibitors of IDO and TDO may be more effective. A subsequent study has demonstrated that navoximod, a dual IDO1/ TDO inhibitor, induces a powerful antitumor immune response and inhibits tumor progression when combined with anti-PD-L1 antibodies [151].

#### Amino acid metabolism-targeting therapy combined with CAR-T/CAR-NK therapy

Inadequate persistence of CAR-T cells in vivo leads to poor therapeutic outcomes and disease recurrence [215]. To optimize the in vitro CAR-T/NK cell expansion process for better clinical efficacy, two main aspects of combination therapy may be considered: one is to enhance the adaptability of immune cells to the TME, and the other is to enhance the cytotoxicity of immune cells.

In the TME, nutrient deficiency, accumulation of large amounts of toxic metabolites, hypoxia, and low PH create a unique environment that promotes tumor growth and suppresses immunity. In combination with these two factors, CAR-T/NK cells have difficulty generating adaptive and durable antitumor responses. It has been found that poor proliferation and persistence of T cells is one of the main reasons why adoptive cell therapy has no or a weak response [216]. Therefore, in vitro cultured immune cells also require potent metabolic capacity to enhance their adaptability to harsh environments. The upregulation of amino acid transporters SLC1A5 and SLC7A5 may improve the function of CAR-NK and CAR-T cells [28]. In a recent study, authors found that re-engineering CAR-T cells to express SLC7A5 or SLC7A11 can promote CAR-T cells proliferation and IFN-γ release under low tryptophan or cystine conditions [217]. Thus, loading CAR-T/NK cells with amino acid transporters may enhance their resilience to amino acid depletion and improve their function. Another approach is to consider the loading of functional amino acid-metabolizing enzymes. Because of the low expression of argininosuccinate synthase and ornithine transcarbamylase, T cells are susceptible to arginine depletion [46]. Thus, T cells can be reengineered to express functional argininosuccinate synthase or ornithine transcarbamylase enzymes in conjunction with different CARs, which increases CAR-T cell proliferation without reducing cytotoxicity.

Another major strategy is to enhance immune cytotoxicity. This can be achieved through simultaneous or sequential application of drugs that directly target amino acid metabolism. For example, CD19 CAR-T had no effect on IDO-positive tumors but was restored in combination with 1-MT [20]. In contrast, CAR-T cell cytotoxicity can be enhanced by regulating T cell metabolism during in vitro expansion. In vitro-amplified CAR-T cells showed phenotypic heterogeneity, most of which were effector memory T cell or effector T cell subpopulations, and naïve T cell and central memory T cell populations, which showed stronger cytotoxicity, were very low. Transformation of differentiated subsets is closely related to the metabolic adaptability of T cells. One study demonstrated that pretreatment of CAR-T/NK cells with the glutamine antagonist 6-diazo-5-oxygen-L-deamine in vitro modulated the differentiation phenotype and enhanced metabolic adaptability [215]. Similarly, a recent study found that dynamic in vitro culture can enhance the antitumor activity of immune cells [218]. Dynamic culture can increase glutamine metabolic flux and promote ATP production. These cells are in a high metabolic state to produce increased amounts of energy. These findings provide new insights into the expansion of immune cells in vitro. However, it is worth noting that over-enhancing the cytotoxicity of CAR-T may produce toxic levels of cytokines and over-activation of immune system, leading to cytokine release syndrome or neurotoxicity [216]. Therefore, it is very important to have the right window of treatment. With the continuous optimization of CAR molecules and the development of combination therapy, it is believed that CAR-T cell therapy will be safer and more efficient.

## Conclusions

In recent years, significant progress has been made in amino acid metabolism reprogramming in the TME. As an increasing number of mechanisms have been elucidated, targeting amino acid metabolism opens up new avenues for the treatment of cancer patients. Tumor control by targeting various stages of amino acid metabolism, including the inhibition of amino acid uptake, transport, and metabolism, has demonstrated to be a promising therapeutic strategy. In addition, the presence of complex crosstalk between amino acid metabolism and T cells in the TME is becoming clear, which may determine the fate of T cells and play a considerable role in immune escape in tumors. In this regard, the limited amino acids in the TME and high metabolic activity of tumor cells result in nutrient competition between tumor cells and T cells and produce a large number of toxic metabolites. Furthermore, complex metabolic crosstalk between amino acids, glucose, and lipids can influence T cell immunity. The profound significance of amino acid metabolism in T cells has made it a popular topic in oncotherapy. Targeting amino acid metabolism combined with ICB or adoptive cell therapy can significantly enhance the efficacy of immunotherapy by strengthening the effector functions of T cells.

In conclusion, targeting amino acid metabolism is a promising therapeutic strategy; however, many challenges remain to be addressed. For example, because drugs are usually administered throughout the body, targeting amino acid metabolism will inevitably cause toxic side effects. Even if the drug reaches the tumor site, there is no guarantee that the drug can target the tumor with high specificity and without affecting the antitumor immune cells. In addition, the plasticity of tumor cell metabolism and differences in amino acid dependence make it difficult to select drugs. Therefore, how can we target tumor cell metabolism while avoiding the toxic effects on immune and normal cells? How do we define the metabolic subtypes of tumor cells? How do we prevent tumors from developing resistance to targeted metabolic drugs?

In recent years, with the vigorous development of metabolomics, such as high-resolution mass spectrometry [219], determining the metabolic subtypes of tumors is possible, which is conducive to individualized targeted therapy. However, targeted amino acid metabolism in combination with immunotherapy has made significant breakthroughs in both preclinical and clinical trials, promising to overcome the limits of treatment for patients with advanced cancer. To date, several highly specific small-molecule inhibitors targeting amino acid metabolic pathways, such as the IDO inhibitor BM-986205, ARG inhibitor CB-1158, and kynureninase, have been evaluated in multiple clinical trials as monotherapy or in combination with ICB [37]. Future research will help reveal key features of amino acid metabolism in the TME based on T cell immunity, which will provide important insights into the design of effective drugs targeting amino acid metabolism and combining with immunotherapy.

## Data Availability

Not applicable.

## Abbreviations

TME: Tumor microenvironment
TCA cycle: Tricarboxylic acid cycle
ATP: Adenosine-triphosphate
ALL: Acute lymphoblastic leukemia
ICB: Immune checkpoint blockade
PD-1: Programmed cell death protein 1
PD-L1: Programmed cell death 1 ligand 1
CTLA-4: Cytotoxic T lymphocyte-associated antigen-4
CAR-T: Chimeric antigen receptor-T
CAR-NK: Chimeric antigen receptor-natural killer
IDO: Indoleamine 2, 3-dioxygenase
mTOR: Mammalian target of rapamycin
mTORC1: MTOR complex 1
mTORC2: MTOR complex 2
S6K1: P70 S6 kinase
4E-BP1: EIF4E-binding protein
ODC: Ornithine decarboxylase
Tregs: Regulatory T cells
GCN2: General control nonderepressible 2
eIF2α: Eukaryotic initiation factor 2
CDK4: Cyclin-dependent kinase 4
TCR: T-cell receptor
IFN-γ: Interferon-γ
ATF4: Activating transcription factor 4
PGE2: Prostaglandin E2
GSH: Glutathione
DCs: Dendritic cells
FOXO: Forkhead box O
PTEN: Phosphatase with tensin homology
PI3K: Phosphatidylinositol 3-kinase
PTMs: Post-translational modifications
SAM: S-adenosyl methionine
α-KG: α-Ketoglutaric acid
eIF5A: Eukaryotic initiation factor 5A
NO: Nitric oxide
ONOO: Peroxynitrite
UDP-GlcNAc: Uridine diphosphate n-acetyl glucosamine
ROS: Reactive oxygen species
ERK: Extracellular signal-regulated kinase
JNK: C-Jun amino-terminal kinase
Kyn: Kynurenine
3-HK: 3-Hydroxykynurenine
3-HAA: 3-Hydroxyanthranilic acid
TDO: Tryptophan 2, 3-dioxygenase
AhR: Aryl hydrocarbon receptor
ARG: Arginase
iNOS: Inducible nitric oxide synthase
GABA: Gamma-aminobutyric acid
OXPHOS: Oxidative phosphorylation
PKM2: Pyruvate kinase isozymes M2
LDH-A: Lactate dehydrogenase A
GLS2: Glutaminase 2
PEG: Polyethylene glycol
MDSCs: Bone marrow-derived suppressor cells
SLC: Solute carrier
BCH: 2-Aminobicyclo-(2,2,1)-heptane-2-carboxylic acid
CDK: Cyclin-dependent kinase
GPNA: γ-L-glutamyl-p-nitroanilide
α-MT: Alpha-methyltryptophan
COX-2: Cyclooxygenase-2
NOHA: N-hydroxy-l-arginine
nor NOHA: Nω-hydroxyl-non-arginine
GDH: Gutamate deaminase
PHGDH: Phosphate dehydrogenase
